# Effect of low energy availability during three consecutive days of endurance training on iron metabolism in male long distance runners

**DOI:** 10.14814/phy2.14494

**Published:** 2020-06-29

**Authors:** Aya Ishibashi, Chihiro Kojima, Yoko Tanabe, Kaito Iwayama, Tsutomu Hiroyama, Toshiki Tsuji, Akiko Kamei, Kazushige Goto, Hideyuki Takahashi

**Affiliations:** ^1^ Japan Institute of Sports Sciences Kitaku Tokyo Japan; ^2^ Department of Life Sciences The University of Tokyo Meguro Tokyo Japan; ^3^ Faculty of Health and Sport Sciences University of Tsukuba Tsukuba Ibaraki Japan; ^4^ Department of Budo and Sport Studies Tenri University Tenri Nara Japan; ^5^ Graduate School of Sport and Health Science Ritsumeikan University Kusatsu Shiga Japan

**Keywords:** endurance runner, energy availability, iron metabolism, muscle glycogen

## Abstract

We investigated the effect of low energy availability (LEA) during three consecutive days of endurance training on muscle glycogen content and iron metabolism. Six male long distance runners completed three consecutive days of endurance training under LEA or neutral energy availability (NEA) conditions. Energy availability was set at 20 kcal/kg fat‐free mass (FFM)/day for LEA and 45 kcal/kg FFM/day for NEA. The subjects ran for 75 min at 70% of maximal oxygen uptake ( $\dot{V}$O_2max_) on days 1–3. Venous blood samples were collected following an overnight fast on days 1–4, immediately and 3 hr after exercise on day 3. The muscle glycogen content on days 1–4 was evaluated by carbon‐magnetic resonance spectroscopy. In LEA condition, the body weight and muscle glycogen content on days 2–4, and the FFM on days 2 and 4 were significantly lower than those on day1 (*p* < .05 vs. day1), whereas no significant change was observed throughout the training period in NEA condition. On day 3, muscle glycogen content before exercise was negatively correlated with serum iron level (immediately after exercise, 3 hr after exercise), serum hepcidin level immediately after exercise, and plasma IL‐6 level immediately after exercise (*p* < .05). Moreover, serum hepcidin level on day 4 was significantly higher in LEA condition than that in NEA condition (*p* < .05). In conclusion, three consecutive days of endurance training under LEA reduced the muscle glycogen content with concomitant increased serum hepcidin levels in male long distance runners.

## Graphical abstract

Three consecutive days of endurance training under low energy availability (LEA) decreased the muscle glycogen content and increased the serum hepcidin level in well‐trained male long distance runners. These results suggest that LEA is associated with a risk of exercise‐induced iron deficiency by an elevated hepcidin level in endurance athletes.

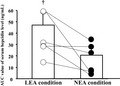

## 1 INTRODUCTION

Iron deficiency is a highly prevalent nutritional disorder among endurance athletes (Beard & Tobin, 2000). Several physiological mechanisms have been proposed to explain impaired iron status in athletes, including gastrointestinal bleeding, hemolysis, lack of iron in diet, and iron loss in sweat. In addition, an iron regulatory hormone hepcidin may act as another potential factor contributing to iron deficiency (Peeling et al., 2009). Hepcidin, a 25‐amino acid peptide, is a crucial mediator of iron homeostasis (Ganz & Nemeth, 2006). Hence, hepcidin may be related to iron deficiency in response to exercise training.

Prolonged exercise causes marked increase in the circulating level of the cytokine interleukin‐6 (IL‐6), which is suggested as a causal mechanism for elevated hepcidin levels following the exercise (Banzet et al., 2012). In addition, reduction of the muscle glycogen content has been shown to increase exercise‐induced IL‐6 (Keller et al., 2001). Additionally, carbohydrate (CHO) ingestion influenced IL‐6 mRNA and plasma cytokine levels after a 3‐hr run (Nieman 2003), and nutritional interventions attenuated the IL‐6 response to exercise (Hennigar, McClung & Pasiakos 2017). Thus, CHO supplementation during prolonged exercise may mitigate the exercise‐induced elevation of the IL‐6 level. The influence of manipulation of CHO intake (3 vs. 10 g/kg BW) after a glycogen‐depleting run on the exercise‐induced hepcidin response on the following day was previously investigated. Consequently, on the following day, a low‐CHO trial (consumption of a low‐CHO meal after a glycogen‐depleting run) showed significant higher plasma IL‐6 level during postexercise and serum hepcidin levels pre‐exercise (Badenhorst et al., 2015). Furthermore, low energy intake (EI) and low energy balance have been associated with an elevated hepcidin level after 4 days of military training (Pasiakos et al., 2016). Therefore, reduced EI, in particular lowered CHO intake, during endurance period training may promote elevation of the hepcidin level.

Low energy availability (LEA), mediated by a reduced EI and/or increased EEE, develops undesirable statuses, including impaired resting energy expenditure, disruptions of an array of hormonal, metabolic and functional characteristics (Mountjoy, Burke, Stellingwerff, & Sundgot‐Borgen, 2018). Therefore, low energy availability is thought to facilitate relative energy deficiency in sport (RED‐S). RED‐S occurs in both genders and decreases endurance capacity, increases the risk of injury, and reduces glycogen stores (Statuta, Asif, & Drezner, 2017). In fact, LEA is frequently observed in endurance athletes (e.g., marathon runners) during daily training (Loucks, 2007), and it may increase the risk of iron deficiency (Petkus, Murray‐Kolb, & De Souza, 2017). However, the influence of LEA during endurance training period on iron metabolism, particularly the hepcidin level, is unclear.

Therefore, we investigated the impact of LEA during three‐consecutive days of endurance training on the muscle glycogen content and iron metabolism in male long‐distance runners. We hypothesized that LEA during the endurance training period would increase the IL‐6 and hepcidin levels with decreased muscle glycogen content.

## 2 MATERIALS AND METHODS

### 2.1 Subjects

Six well‐trained male long distance runners participated in this study (mean ± standard error [*SE*]: age, 19.8 ± 0.4 years; height, 1.74 ± 0.12 m; body weight [BW], 59.9 ± 1.7 kg; and $\dot{V}$O_2max_, 67.9 ± 1.5 ml kg^−1^ min^−1^). Their average 5,000 m record was 15 min 18 ± 18 s. All subjects trained regularly (6 days/week, 4 hr/day). The exclusion criteria were smoking and use of herbal medicines, supplements, and medications. None of the subjects have taken iron supplements before and during the experiment.

After being informed regarding the study protocols, benefits, and risks, a written informed consent was obtained from all subjects. The study was approved by the Ethics Committee for Human Experiments of the Japan Institute of Sports Sciences (JISS‐IRB‐2016‐050).

### 2.2 Experimental design

The present study used a randomized crossover design, and the subjects performed exercise under LEA or neutral energy availability (NEA) conditions. The EI and EEE were strictly controlled during LEA (<20 kcal/kg fat‐free mass [FFM]/day) condition and NEA (>45 kcal/kg FFM/day) condition (Fagerberg, 2018; Koehler et al., 2016). Both NEA and LEA conditions consisted of four‐consecutive days, including three‐consecutive days of endurance training (days 1–3) and post‐training measurement on the following day (day 4). All measurements were conducted at the Japan Institute of Sports Sciences (JISS). All meals were provided and all training sessions were completed under rigorous supervision. Each condition was repeated in a randomized order, with at least 1 week in‐between as a washout period. The changes in body weight, muscle glycogen content, and blood parameters were determined during the endurance training and post‐training day (days 1–4, Figure 1).

**FIGURE 1:**
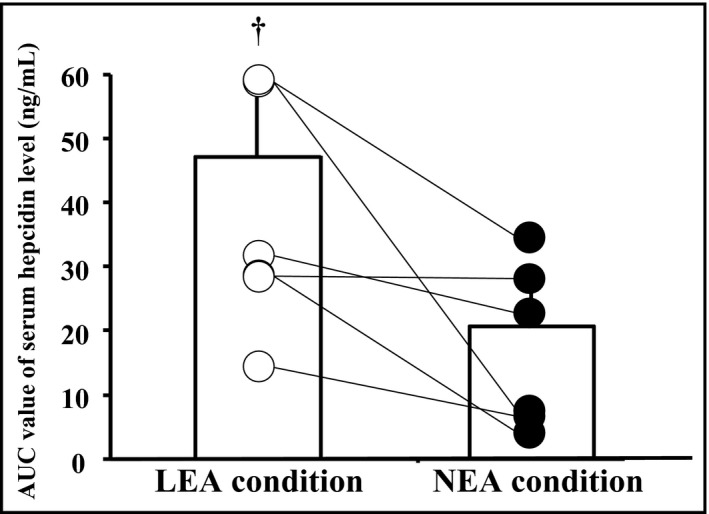
Average and individual area under the curve (AUC) values of the serum hepcidin level during days 1–4. ^†^Significant difference from NEA (*p* < .05). LEA, low energy availability; NEA, neutral energy availability.

### 2.3 Three consecutive days of endurance training

The subjects completed 75 min of running on a treadmill (Pulsar^®^3p; h/p/Cosmos Sports and Medical Gmbh) during days 1–3 (1,000–1,130). The running velocity was set at 70% of the $\dot{V}$O_2max_. Water was available ad libitum throughout all sessions.

### 2.4 Manipulation of energy availability

The EA was defined as dietary EI minus exercise energy expenditure (EEE). Because energy expenditure in FFM is greater than that in fat mass (FM), the EA is generally normalized to the FFM [EA = (EI − EEE) ÷ FFM] (Loucks, 2004). The EEE was calculated from $\dot{V}$O_2_ and $\dot{V}$CO_2_ using the Weir equation (Weir, 1949). The EEE was also determined by subtracting the resting metabolic rate (RMR) from the EEE.

$$\text{EEE}\left(\text{kcal}/\min\right)\;=\;\left(3.9\;\times \;{\dot{V}O}_{2}+1.1\;\;\times \;{\dot{V}\text{CO}}_{2}\right)\;\times \;0.7-\text{RMR}$$

### 2.5 Experimental protocol

#### 2.5.1 Maximal oxygen uptake

The running velocity corresponding to 70% of an individual's $\dot{V}$O_2max_ was determined from the regression between $\dot{V}$O_2_ and running velocity during an incremental exercise test on a treadmill (Ohtake Root). The test was initiated at 12.6 or 13.8 km/hr and the running velocity was increased by 1.2 km/hr every 3 min. After completing six submaximal stages, the subjects restarted running after a 5 min rest from 18.6 or 19.8 km/hr, and the running velocity was increased 0.6 km/hr every 1 min until voluntary exhaustion to determine the $\dot{V}$O_2max_. Respiratory gas was continuously collected by a breath‐by‐breath method using the computerized standard open‐circuit technique (AE310S; Minato Medical Science Co.).

#### 2.5.2 Diet prescriptions

All food and water consumed during the present study was provided and recorded by the JISS Sports Nutrition team. On days 1–3, meals were individually planned by a nutritionist, and cooked meals were provided three times in a buffet restaurant at JISS (0830 for breakfast, 1,200–1,300 for lunch, and 1,800 for dinner). The estimated EEE from their training was 1,117 ± 41 kcal/day, and the EI was calculated to match required LEA (EA < 20 kcal/kg FFM/day) and NEA (EA > 45 kcal/kg FFM/day). All subjects who had leftover food were required to report to a nutritionist, and their dietary prescriptions and adherence to reporting requirements were checked during training period. The subjects were allowed to consume water ad libitum, but they were instructed to avoid eating and drinking aside from the prescribed meal plans. During the experimental period, we monitored the BW of the subjects so that the BW did not change rapidly.

#### 2.5.3 Body composition

The BM, FFM, and FM were evaluated using a multifrequency impedance technique (InBody720; Biospace, South Korea). Using a range of frequencies from 1 kHz to 1 MHz, the InBody720 accurately measures the amount of body water and body composition (Demura, Sato, & Kitabayashi, 2004). The subjects emptied their bladders prior to the measurements (before breakfast, 0730–0800).

#### 2.5.4 Blood sampling and analyses

Resting blood samples were collected from an antecubital vein within 1min after an overnight fast on days 1–4 (at least 12 hr after the last meal, 0,800 – 0,830). On day 3, further blood samples were collected immediately (1,130) and 3 hr (1,430) after the exercise session to evaluate exercise‐induced IL‐6 and hepcidin elevations. All blood tests were performed at room temperature (20–23°C).

For the resting blood samples, 2 ml of blood was transferred to ethylenediaminetetraacetic acid‐containing tubes immediately after blood drawing for determination of the hemoglobin (Hb) level at a clinical laboratory (LSI Medience Co.). The remaining samples were centrifuged (3,000 rpm, 4°C, 10 min), and serum and plasma were stored at −80°C. The plasma or serum were stored in airtight boxes using‐cryofreeze tubes with screw caps, which allowed for a longer storage period (Hackney & Viru, 2008). The serum ferritin and iron levels were measured at the same clinical laboratory. The plasma IL‐6 and serum hepcidin levels were determined using a commercial enzyme‐linked immunosorbent assay kit (R&D Systems Inc.). The intra‐assay coefficients of variation (CV) were 5.2% for IL‐6 and 3.4% for hepcidin.

#### 2.5.5 Muscle glycogen content

Muscle glycogen content was measured noninvasively using carbon‐magnetic resonance spectroscopy (^13^C‐MRS). The ^13^C‐glycogen signal was obtained from the triceps muscle of the right leg using a 3T magnetic resonance system (Magnetom Verio and Skyra; Siemens, Germany) with a ^13^C‐^1^H double‐tuned surface coil 10 cm in diameter. The ^13^C‐glycogen signal was obtained in 15‐min blocks (4,500 scans with a repetition time of 200 msec). Quantification of the ^13^C‐glycogen signal was performed by phantom replacement measurements using a phantom with 120 mM glycogen and 50 mM KCl. These procedures were established in previous study (Takahashi et al., 2015). The peak area of the ^13^C‐glycogen signal was integrated using the software bundled with the MR console. The CV of repeated measurements of muscle glycogen content by ^13^C‐MRS, with repositioning and reshimming, was 3.5 ± 2.1% (Takahashi et al., 2015).

### 2.6 Statistical analyses

Data are presented as means ± *SE*. The normality of the data distribution was initially assessed by the Shapiro–Wilk test. Changes in blood variables over time were evaluated by two‐way analysis of variance (ANOVA) with repeated measures (1: condition [LEA, NEA] ×time [days 1–4], 2: condition [LEA, NEA] × time [pre‐exercise, postexercise, and/or 3 hr after post‐exercise on day 3]). When the ANOVA revealed a significant interaction or main effect, Bonferroni *post‐hoc* analysis was performed. The area under the curve (AUC) of the serum hepcidin level was calculated during days 1–4. An independent *t*‐test was used to compare the AUC in LEA and NEA conditions.

We calculated Cohen's *d*‐values *d*‐values or partial η^2^ values to determine effect sizes. All analyses were performed using SPSS software (ver.24.0; IBM Corp.). A *p*‐value of <.05 was considered to reflect statistical significance.

## 3 RESULTS

### 3.1 Running distance and energy expenditure during training sessions

The average values of running distance and EEE during training sessions were 19.0 ± 0.4 km/day (NEA condition) and 18.9 ± 0.5 km/day (LEA condition). There was no significant difference in either of these variables between NEA and LEA conditions (*p* > .05).

### 3.2 EI, macronutrients, and EA during the training period

Table 1 presents the EI and EA on days 1–3. LEA condition showed significantly lower EI, macronutrients and EA compared to NEA condition. The average EA during days 1–3 was <20 kcal/kg FFM/day in LEA condition and >45 kcal/kg FFM/day in NEA condition. The daily iron intake on days 1–3 did not differ significantly between NEA and LEA conditions (NEA condition, 11.1 ± 1.3 mg/day; LEA condition, 9.0 ± 0.4 mg/day; *p* > .05).

**TABLE 1:** Total EI and macronutrients intake during training period

	Condition	Day 1	Day 2	Day 3	Interaction (partial η^2^)	Condition (partial η^2^)	Time (partial η^2^)
EI (kcal)	NEA	3,904 ± 68	3,915 ± 75	4,112 ± 164	0.21 (0.14)	<0.01 (0.97)	0.20 (0.15)
	LEA	2,042 ± 75^†^	2,099 ± 68^†^	2,071 ± 70^†^			
Protein (g)	NEA	158.8 ± 4.6	158.8 ± 5.9	160.6 ± 7.7	0.27 (0.12)	0.01 (0.82)	0.24 (0.13)
	LEA	100.5 ± 8.1^†^	114.7 ± 4.4^†^	109.1 ± 6.4^†^			
Fat (g)	NEA	117.7 ± 9.5	116.3 ± 8.7	118.0 ± 5.5	0.62 (0.04)	<0.01 (0.86)	0.73 (0.03)
	LEA	52.4 ± 4.1^†^	58.7 ± 3.6^†^	57.6 ± 5.5^†^			
CHO (g)	NEA	544.3 ± 14.2	548.6 ± 14.4	589.7 ± 23.7	0.23 (0.14)	<0.01 (0.99)	0.42 (0.31)
	LEA	288.1 ± 10.0^†^	277.5 ± 7.4^†^	276.5 ± 16.7^†^			
CHO/BW (g/kg)	NEA	9.2 ± 0.6	9.0 ± 0.4	9.6 ± 0.3	0.42 (0.08)	<0.01 (0.94)	0.51 (0.06)
	LEA	4.8 ± 0.2^†^	4.6 ± 0.2^†^	4.6 ± 0.3^†^			
EA (kcal/FFM)	NEA	51.2 ± 2.4	51.3 ± 2.6	54.7 ± 2.3	0.16 (0.17)	<0.01 (0.96)	0.41 (0.08)
	LEA	17.3 ± 0.6^†^	18.9 ± 0.9^†^	19.1 ± 1.0^†^			

Note

Values are means ± *SE*. *p* ^†^, Significant difference from NEA condition.

BW, body weight; CHO, carbohydrate; EI, energy intake (*p* < .05); LEA, low energy availability; NEA, neutral energy availability.

### 3.3 Body composition during the training period

Table 2 presents the body composition data for days 1–4. The BW and FFM exhibited a significant interaction. In LEA condition, the BW on days 2–4 and the FFM on day 2 and 4 were significantly lower than those on day 1, whereas these parameters did not change significantly in NEA condition. The FM did not differ significantly between NEA and LEA conditions during the training period.

**TABLE 2:** Body weight, lean body mass, skeletal muscle, and fat mass (resting levels) on days 1–4

	Condition	Day 1	Day 2	Day 3	Day 4	Interaction (partial η^2^)	Condition (partial η^2^)	Time (partial η^2^)
BW (kg)	NEA	61.8 ± 2.4	62.2 ± 2.5	62.1 ± 2.5	62.1 ± 2.5	0.78 (0.01)	0.78 (<0.01)	0.01 (0.31)
	LEA	61.8 ± 2.3	61.0 ± 2.4*	61.0 ± 2.4*	60.6 ± 2.4*			
FFM (kg)	NEA	55.8 ± 2.1	56.2 ± 2.3	55.9 ± 2.2	56.3 ± 2.3	0.04 (0.27)	0.86 (<0.01)	0.64 (0.05)
	LEA	56.0 ± 2.1	55.1 ± 2.3*	55.6 ± 2.3	55.1 ± 2.3*			
FM (kg)	NEA	6.1 ± 0.5	6.0 ± 0.4	6.2 ± 0.4	5.8 ± 0.4	0.40 (0.09)	0.50 (0.05)	0.44 (0.09)
	LEA	5.7 ± 0.5	5.8 ± 045	5.4 ± 0.4	5.5 ± 0.4			

Note

Values are means ± *SE*.

Abbreviations: BW, body weight; FFM, fat free mass; FM, fat mass; LEA, low energy availability; NEA, neutral energy availability.

* Significant difference from resting on day 1 (*p* < .05).

### 3.4 Hepcidin, iron parameter and IL‐6 

#### 3.4.1 Hepcidin

Table 3 presents the serum hepcidin level (resting level) on days1–4; there was a significant interaction, and significant main effects of condition and time. On day 2 in LEA condition, the serum hepcidin level was significantly higher than on day1, whereas it did not change significantly in NEA condition. Moreover, the AUC over days 1–4 was significantly higher in LEA condition than in NEA condition (*p* = .047, *d* = 1.25, Figure 1).

On day 3, the serum hepcidin level was significantly elevated 3 hr after exercise in LEA and NEA conditions (NEA condition: pre‐exercise, 5.7 ± 1.8 ng/ml; 3 hr post‐exercise, 20.6 ± 6.7 ng/ml; LEA condition: pre‐exercise, 13.0 ± 3.4 ng/ml; postexercise 34.9 ± 9.4 ng/ml). However, these responses were not significantly different between LEA and NEA conditions (interaction [condition × time], *p* = .665 [partial η^2^ = 0.02]; condition, *p* = .135 [partial η^2^ = 0.21]; time, *p* = .001 [partial η^2^ = 0.66]).

#### 3.4.2 Iron parameter and IL‐6

The blood Hb level did not differ significantly between LEA and NEA conditions (NEA: 14.3 ± 0.5 g/dl on day 1, 13.8 ± 0.3 g/dl on day 4; LEA: 14.6 ± 0.2 g/dl on day 1, 14.2 ± 0.3 g/dl on day 4). As shown in Table 3, serum ferritin level did not exhibit significant interaction or a main effect of time. However, the serum ferritin level was significantly higher in LEA condition on day 4. The serum iron level on days 1–4 did not exhibit a significant interaction or main effect of condition, but a significant main effect of time was observed.

**TABLE 3:** Iron parameter, hepcidin and IL‐6 (resting level) during training period on days 1–4

	Condition	Day 1	Day 2	Day 3	Day 4	Interaction (partial η^2^)	Condition (partial η^2^)	Time (partial η^2^)
Hepcidin (ng/ml)	NEA	8.5 ± 2.6	8.8 ± 4.6	6.1 ± 2.7	6.0 ± 3.1	0.337 (0.24)	0.080 (0.28)	0.003 (0.37)
	LEA	8.9 ± 2.6	22.2 ± 4.6*	13.0 ± 2.7	14.4 ± 3.1^†^			
Ferritin (ng/ml)	NEA	40.1 ± 8.7	‐‐‐	‐‐‐	40.2 ± 9.6	0.01 (0.51)	1.00 (0.01)	0.04 (0.35)
	LEA	35.2 ± 6.5	‐‐‐	‐‐‐	56.6 ± 9.9*			
Iron	NEA	148 ± 38	143 ± 23	99 ± 6	132 ± 7	0.74 (0.04)	0.88 (<0.01)	<0.01 (0.37)
(μg/dl)	LEA	119 ± 68	157 ± 44*	131 ± 30	157 ± 14*			
IL‐6	NEA	0.14 ± 0.04	0.08 ± 0.03	0.15 ± 0.07	0.10 ± 0.03	0.458 (0.11)	0.153 (0.28)	0.157 (0.30)
(pg/ml)	LEA	0.23 ± 0.09	0.30 ± 0.12	0.19 ± 0.04	0.19 ± 0.07			

Note

Values are means ± *SE*.

* Significant difference from resting on day 1 (*p* < .05).

^†^ Significant difference from NEA condition (*p* < .05).

On day 3, the serum iron level did not exhibit a significant interaction or main effect of condition after exercise (LEA condition: pre‐exercise, 178.8 ± 15.8 μg/dl, 3 hr postexercise 152.8 ± 16.1 μg/dl; NEA condition, pre‐exercise: 157.6 ± 8.0 μg/dl, 3 hr after exercise: 129.6 ± 8.1 μg/dl).

Table 3 presents the plasma IL‐6 level (resting level) on days 1–4; there was no significant interaction or main effect of condition, but a significant main effect of time was observed. On day 3, the plasma IL‐6 level was significantly elevated immediately after exercise, and the exercise‐induced elevation of the IL‐6 level was greater in LEA condition than in NEA condition [NEA condition: pre‐exercise, 0.15 ± 0.07 pg/ml; postexercise, 0.39 ± 0.11 pg/ml; LEA condition: pre‐exercise, 0.19 ± 0.04 pg/ml; post‐exercise, 1.36 ± 0.75 pg/ml; interaction (condition × time), *p* = .039 (partial η^2^ = 0.36); condition, *p* = .011 (partial η^2^ = 0.49); time, *p* = .022 (partial η^2^ = 0.24)].

### 3.5 Muscle glycogen content

Figure 2 shows the muscle glycogen content of the triceps surae muscle on days 1–4. The muscle glycogen content exhibited a significant interaction. On days 2–4 in LEA condition, the muscle glycogen content was significantly lower than that on day1, whereas it did not change significantly in NEA condition. Thus, there were significant differences in muscle glycogen content between the two conditions from days 2–4.

**Figure 1:**
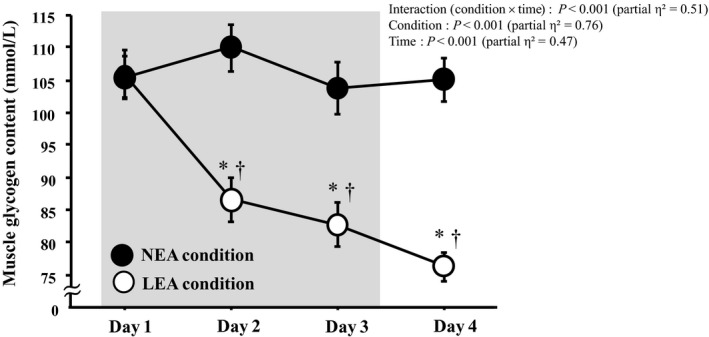
Muscle glycogen content during the training period on days 1–4. Values are means ± SE. *Significant difference from resting on day 1 (p < .05). †Significant difference from NEA (p < .05). LEA, low energy availability; NEA, neutral energy availability, Gray box indicates the endurance training period

## 4 DISCUSSION

The major finding of the present study was that three consecutive days of endurance training under LEA significantly increased the resting serum hepcidin levels with concomitant reduction of muscle glycogen content However, elevated hepcidin was not associated with inflammation. Meanwhile lowered muscle glycogen content during training period was associated with exercise‐induced elevations of plasma IL‐6, serum iron, and hepcidin levels. Notably, five of six subjects had a higher serum hepcidin level in LEA condition than in NEA condition during training period, whereas only a subject had a slightly (1.1%) higher hepcidin level in NEA condition.

Serum hepcidin levels are generally elevated after a single bout of exercise, and the exercise‐induced elevations are influenced by exercise intensity and duration in athletes (Peeling et al., 2009). Moreover, serum hepcidin levels appear to be affected by energy balance (Pasiakos et al., 2016 and Badenhorst, Black, & O’Brien, 2019). Muscle glycogen content may increase the hepcidin level, possibly due to increased IL‐6 production (Keller et al., 2001). Therefore, increased CHO intake attenuated the elevation of the hepcidin level (Badenhorst et al., 2015). On the other hand, three weeks of manipulations of CHO and fat intakes under well‐controlled dietary intervention study did not affect serum hepcidin levels in elite athletes (McKay et al. 2019). Currently, whether either low energy or low carbohydrate influences the hepcidin elevation is not conclusive. In the present study, we observed significant difference between AUC of resting hepcidin level and the muscle glycogen content over days 1–4. This finding may suggest that lowered muscle glycogen content was related to the elevated hepcidin level following 3‐days of endurance training under LEA. Costill & Miller (1980) reported that subjects who maintained a relatively low‐CHO diet showed a sustained reduction in muscle glycogen content during consecutive days of endurance training. When the same subjects consumed a high‐CHO diet during the training period, the muscle glycogen content recovered to the baseline level within 24 hr postexercise. A CHO intake of 10–12 g kg^−1^ day^−1^ is currently recommended during high‐intensity or high‐volume training (Thomas, Erdman, & Burke, 2016). Indeed, in NEA condition (with consuming CHO about 9 g kg^−1^ day^−1^), the muscle glycogen content did not change significantly throughout days 1–4. A beneficial effect of CHO intake during intensive training on muscle glycogen content has been fully recognized, but our findings suggest another role of sufficient CHO intake, (i.e., maintaining the serum hepcidin level and preventing iron deficiency) for iron metabolism in endurance athletes.

A previous study revealed that gluconeogenic signals regulated iron homeostasis via hepcidin in starving mice (Vecchi et al. 2014). In the present study, serum hepcidin level appears to be increased primarily through altered metabolic signal rather than inflammation. Under low energy availability, hepcidin prevents the growth of pathogenic microorganisms by reducing iron (Andrews, 2004). In this way, elevated hepcidin supports gluconeogenesis in the liver during energy restriction. Therefore, LEA even for a short period of training could alter iron metabolism and increase the risk of iron deficiency in athletes.

On day 3, the exercise‐induced plasma IL‐6 elevation was significantly greater in LEA condition than in NEA condition. A depleted muscle glycogen store facilitates IL‐6 production by contracting muscles (Keller et al., 2001). Muscle‐derived IL‐6 acts as a hormone and it is involved in glucoregulatory processes during endurance exercise (Kanemaki et al., 1998). In addition, the exercise‐induced increase in plasma IL‐6 level has been suggested to stimulate hepcidin production (Banzet et al., 2012). Therefore, in LEA condition, increased serum hepcidin level and plasma IL‐6 levels 3 hr after exercise on day 3 may be explained by reduced muscle glycogen content. In contrast, the serum hepcidin level 3 hr after exercise on day 3 did not differ significantly between LEA and NEA condition, despite different IL‐6 levels. Peeling et al. (2017) reported a weak correlation between the exercise‐induced elevations of the IL‐6 and hepcidin levels in endurance athletes.

The present study includes several limitations. First, the sample size was small, because we recruited only highly trained long‐distance runners and without iron deficiency anemia. The subjects were involved in strictly controlled laboratory‐based experiment with conditions and a randomized cross‐over design. They were members of the same team and maintained similar training schedules during the experiment, including the washout period between LEA and NEA conditions. Despite small sample size, the comparisons of AUC and the change in serum hepcidin levels between the two conditions presented apparent differences. We also calculated the effect size (d), for the AUC of serum hepcidin level over days 1–4, and it revealed that the difference was “large” (*d* = 1.25). Furthermore, five of six subjects presented a greater AUC of the serum hepcidin level under LEA condition than under NEA condition. Therefore, we think that the present result (i.e., elevated serum hepcidin levels in LEA condition) is robust. Second, the findings may be specific to relatively short‐term endurance training. We used the short‐term (3 days) endurance training condition to mimic the rapid increase in training stress associated with a short‐duration training camp. Moreover, because we adjusted all meals during days 1–3 on an individual basis to meet the EI requirement, monitoring during long‐term training would have been impractical in the laboratory setting. Therefore, further studies are needed to explore whether our findings are applicable to long‐term intensive training in the real situation. Also, we were unable to distinguish between the impact of low EI and low CHO intake on the elevated serum hepcidin level under LEA. In general, endurance athletes under LEA have insufficient CHO intake (Loucks, 2004). Heikura, Stellingwerff, Mero, Uusitalo, & Burke (2017) reported that 26% of elite long distance runners regularly restricted their CHO intake. Thus, we speculate that the elevated serum hepcidin level under LEA is mediated by CHO shortage. Likewise, we evaluated only the resting muscle glycogen content, and exercise‐induced muscle glycogen utilization was not determined. In future studies, the impact of CHO intake during intensified endurance training period on iron metabolism should be clarified.

## 5 CONCLUSION

Three consecutive days of endurance training under LEA decreased the muscle glycogen content and increased the serum hepcidin level in well‐trained male long‐distance runners. These results suggest that LEA is associated with a risk of exercise‐induced iron deficiency by an elevated hepcidin level in endurance athletes.

The findings provide the novel message that maintenance of sufficient EA would reduce the risk of iron deficiency during consecutive days of endurance training period in endurance athletes. Therefore, athletes and coaches should pay attention to energy balance and CHO intake during intensive training period to maintain optimal iron metabolism.

## CONFLICT OF INTEREST

All authors have reported no relevant conflicts of interest.

## References

[phy214494-bib-0001] Andrews, N. C. (2004). Anemia of inflammation: The cytokine‐hepcidin link. The Journal of Clinical Investigation, 113(9), 1251–1253. https://www.ncbi.nlm.nih.gov/pmc/articles/PMC398435/. 10.1172/JCI21441 15124013 PMC398435

[phy214494-bib-0002] Badenhorst, C. E. , Black, K. E. , & O’Brien, W. J. (2019). Hepcidin as a prospective individualised biomarker for individuals at risk of low energy availability. International Journal of Sport Nutrition and Exercise Metabolism, 29(6), 671–681. 10.1123/ijsnem.2019-0006 31034252

[phy214494-bib-0003] Badenhorst, C. E. , Dawson, B. , Cox, G. R. , Laarakkers, C. M. , Swinkels, D. W. , & Peeling, P. (2015). Acute dietary carbohydrate manipulation and the subsequent inflammatory and hepcidin responses to exercise. European Journal of Applied Physiology, 115(12), 2521–2530. 10.1007/s00421-015-3252-3 26335627

[phy214494-bib-0004] Banzet, S. , Sanchez, H. , Chapot, R. , Bigard, X. , Vaulont, S. , & Koulmann, N. (2012). Interleukin‐6 contributes to hepcidin mRNA increase in response to exercise. Cytokine, 58(2), 158–161. 10.1016/j.cyto.2012.01.006 22326661

[phy214494-bib-0005] Beard, J. , & Tobin, B. (2000). Iron status and exercise. The American Journal of Clinical Nutrition, 72(2), 594–597. 10.1093/ajcn/72.2.594S 10919965

[phy214494-bib-0006] Costill, D. L. , & Miller, J. M. (1980). Nutrition for endurance sport: Carbohydrate and fluid balance. International Journal of Sports Medicine, 1(1), 2–14. 10.1055/s-2008-1034623

[phy214494-bib-0007] Demura, S. , Sato, S. , & Kitabayashi, T. (2004). Percentage of total body fat as estimated by three automatic bioelectrical impedance analyzers. Journal of Physiological Anthropology and Applied Human Science, 23(3), 93–99. http://www.ncbi.nlm.nih.gov/pubmed/15187381 10.2114/jpa.23.93 15187381

[phy214494-bib-0008] Fagerberg, P. (2018). Negative consequences of low energy availability in natural male bodybuilding: A review. International Journal of Sport Nutrition and Exercise Metabolism, 28(4), 385–402. 10.1123/ijsnem.2016-0332 28530498

[phy214494-bib-0009] Ganz, T. , & Nemeth, E. (2006). Iron imports. IV. Hepcidin and regulation of body iron metabolism. American Journal of Physiology‐Gastrointestinal and Liver Physiology, 290(2), G199–G203. 10.1152/ajpgi.00412.2005 16407589

[phy214494-bib-0010] Hackenry, A. C. , & Viru, A. (2008). Research methodology: endocrinologic measurements in exercise science and sports medicine. Jounal of Athletic Training., 43(6), 631–639. https://www.ncbi.nlm.nih.gov/pmc/articles/PMC2582556/ 10.4085/1062-6050-43.6.631 PMC2582556 19030142

[phy214494-bib-0011] Heikura, I. A. , Stellingwerff, T. , Mero, A. A. , Uusitalo, A. L. T. , & Burke, L. M. (2017). A mismatch between athlete practice and current sports nutrition guidelines among elite female and male middle‐ and long‐distance athletes. International Journal of Sport Nutrition and Exercise Metabolism, 27(4), 351–360. 10.1123/ijsnem.2016-0316 28338358

[phy214494-bib-0029] Hennigar, S. R. , McClung, J. P. , & Pasiakos, S. M. (2017). Nutritional interventions and the IL‐6 response to exercise. FASEB J, 31(9), 3719–3728. 10.1096/fj.201700080R 28507168

[phy214494-bib-0012] Kanemaki, T. , Kitade, H. , Kaibori, M. , Sakitani, K. , Hiramatsu, Y. , Kamiyama, Y. , … Okumura, T. (1998). Interleukin 1β and interleukin 6, but not tumor necrosis factor α, inhibit insulin‐stimulated glycogen synthesis in rat hepatocytes. Hepatology, 27(5), 1296–1303. 10.1002/hep.510270515 9581683

[phy214494-bib-0013] Keller, C. , Steensberg, A. , Pilegaard, H. , Osada, T. , Saltin, B. , Pedersen, B. K. , & Neufer, P. D. (2001). Transcriptional activation of the IL‐6 gene in human contracting skeletal muscle: Influence of muscle glycogen content. The FASEB Journal, 15(14), 2748–2750. 10.1096/fj.01-0507fje 11687509

[phy214494-bib-0014] Koehler, K. , Hoerner, N. R. , Gibbs, J. C. , Zinner, C. , Braun, H. , De Souza, M. J. , & Schaenzer, W. (2016). Low energy availability in exercising men is associated with reduced leptin and insulin but not with changes in other metabolic hormones. Journal of Sports Sciences, 34(20), 1921–1929. 10.1080/02640414.2016.1142109 26852783

[phy214494-bib-0015] Loucks, A. (2004). Energy balance and body composition in sports and exercise. Journal of Sports Sciences, 22(1), 1–14. 10.1080/0264041031000140518 14974441

[phy214494-bib-0016] Loucks, A. (2007). Low energy availability in the marathon and other endurance sports. Sports Medicine, 37(4–15), 1348–1352. 10.2165/00007256-200737040-00019 17465605

[phy214494-bib-0027] McKay, A. K. A. , Peeling, P , Pyne, D. B. , Welvaert, M. , Tee, N. , Leckey, J. J. , … Burke, L. M. (2019). Acute Carbohydrate Ingestion Does Not Influence the Post‐Exercise Iron‐Regulatory Response in Elite Keto‐Adapted Race Walkers. J Sci Med Sport, 22(6), 635–640. 10.1016/j.jsams.2018.12.015 30630742

[phy214494-bib-0017] Mountjoy, M. L. , Burke, L. M. , Stellingwerff, T. , & Sundgot‐Borgen, J. (2018). Relative energy deficiency in sport: The tip of an iceberg. International Journal of Sport Nutrition and Exercise Metabolism, 28(4), 313–315. 10.1123/ijsnem.2018-0149 29972091

[phy214494-bib-0026] Nieman, D. C. , Davis, J. M. , Henson, D. A. , Walberg‐Rankin, J. , Shute, M. , Dumke, C. L. , & McAnulty, S.L. (2003). Carbohydrate Ingestion Influences Skeletal Muscle Cytokine mRNA and Plasma Cytokine Levels After a 3‐h Run. J Appl Physiol, 94(5), 1917–1925. 10.1152/japplphysiol.01130.2002 12533503

[phy214494-bib-0018] Pasiakos, S. M. , Margolis, L. M. , Murphy, N. E. , McClung, H. L. , Martini, S. , Gundersen, Y. , … McClung, J. P. (2016). Effects of exercise mode, energy, and macronutrient interventions on inflammation during military training. Physiological Reports, 4(11), e12820 10.14814/phy2.12820. 10.14814/phy2.12820 27273884 PMC4908496

[phy214494-bib-0019] Peeling, P. , Dawson, B. , Goodman, C. , Landers, G. , Wiegerinck, E. T. , Swinkels, D. W. , & Trinder, D. (2009). Effects of exercise on hepcidin response and iron metabolism during recovery. International Journal of Sport Nutrition and Exercise Metabolism, 19(6), 583–597. Retrieved from http://www.ncbi.nlm.nih.gov/pubmed/20175428. 10.1123/ijsnem.19.6.583 20175428

[phy214494-bib-0020] Peeling, P. , McKay, A. K. A. , Pyne, D. B. , Guelfi, K. J. , McCormick, R. H. , Laarakkers, C. M. , … Burke, L. M. (2017). Factors influencing the post‐exercise hepcidin‐25 response in elite athletes. European Journal of Applied Physiology, 117(6), 1233–1239. 10.1007/s00421-017-3611-3 28409396

[phy214494-bib-0021] Petkus, D. L. , Murray‐Kolb, L. E. , & De Souza, M. J. (2017). The unexplored crossroads of the female athlete triad and iron deficiency: A narrative review. Sports Medicine, 47(9), 1721–1737. 10.1007/s40279-017-0706-2 28290159

[phy214494-bib-0022] Statuta, S. M. , Asif, I. M. , & Drezner, J. A. (2017). Relative energy deficiency in sport (RED‐S). British Journal of Sports Medicine, 51(21), 1570–1571. 10.1136/bjsports-2017-097700. 28684389

[phy214494-bib-0023] Takahashi, H. , Kamei, A. , Osawa, T. , Kawahara, T. , Takizawa, O. , & Maruyama, K. (2015). ^13^C‐MRS reveals a small diurnal variation in the glycogen content of human thigh muscle. NMR in Biomedicine, 28(6), 650–655. 10.1002/nbm.3298 25881007

[phy214494-bib-0024] Thomas, D. T. , Erdman, K. A. , & Burke, L. M. (2016). Nutrition and athletic performance. Medicine & Science in Sports & Exercise, 48(3), 543–568. 10.1249/MSS.0000000000000852 26891166

[phy214494-bib-0028] Vecchi, C. , Montosi, G. , Garuti, C. , Corradini, E. , Sabelli, M. , Canali, S. , … Pietrangelo, A. (2014). Gluconeogenic signals regulate iron homeostasis via hepcidin in mice. Gastroenterology, 146(4), 1060–1069. 10.1053/j.gastro.2013.12.016 24361124 PMC3989026

[phy214494-bib-0025] Weir, J. (1949). New methods for calculating metabolic rate with special reference to protein metabolism. The Journal of Physiology, 109(1–2), 1–9. http://onlinelibrary.wiley.com/doi/10.1113/jphysiol.1949.sp004363/full. 10.1113/jphysiol.1949.sp004363 15394301 PMC1392602

